# UHPLC-MS/MS for Antipsychotic Drug Monitoring: A Systematic Review of Clinical and Analytical Performance

**DOI:** 10.3390/jcm14217544

**Published:** 2025-10-24

**Authors:** Ciprian-Ionuț Băcilă, Bianca-Maria Macavei, Monica Cornea, Bogdan Ioan Vintilă, Andrei Lomnășan, Claudia Elena Anghel, Andreea Maria Grama, Cristina Elena Dobre, Claudia Marina Ichim, Gabriela Cioca

**Affiliations:** 1 Faculty of Medicine, Lucian Blaga University of Sibiu, 550169 Sibiu, Romania; ciprian.bacila@ulbsibiu.ro (C.-I.B.); bogdan.vintila@ulbsibiu.ro (B.I.V.); claudia.anghel@ulbsibiu.ro (C.E.A.); claudia_marina95@yahoo.com (C.M.I.); gabriela.cioca@ulbsibiu.ro (G.C.); 2 “Dr. Gheorghe Preda” Clinical Psychiatry Hospital of Sibiu, 550082 Sibiu, Romania; andreilomnasan96@gmail.com (A.L.); andreeamaria.grama@ulbsibiu.ro (A.M.G.); 3 Neuroscience Scientific Research Collective, 550082 Sibiu, Romania; 4 County Clinical Emergency Hospital of Sibiu, 550245 Sibiu, Romania; 5 Socola Institute of Psychiatry, 700282 Iasi, Romania; cedobre@yahoo.com

**Keywords:** UHPLC, TDM, antipsychotics, clinical psychiatry

## Abstract

**Background/Objectives**: Therapeutic drug monitoring (TDM) of antipsychotic medications plays an important role in optimizing treatment efficacy, reducing adverse effects, and supporting adherence. While Ultra-High Performance Liquid Chromatography–Tandem Mass Spectrometry (UHPLC–MS/MS) has long been the gold standard for antipsychotic quantification, recent advances in automated platforms and microsampling raise questions about its current clinical practicality. This systematic review evaluated the clinical applicability and analytical performance of UHPLC-based methods for monitoring antipsychotic drugs, focusing on precision, recovery, matrix effects, and suitability across various biological matrices. **Methods**: A systematic search of PubMed, Scopus, and Web of Science was conducted for studies published between 2013 and 2024 involving UHPLC-based quantification of antipsychotics in clinical samples from adult patients. Data on analytical parameters, sample matrices, and study characteristics were extracted. A custom quality checklist was used to assess methodological rigor. In addition to qualitative synthesis, non-traditional quantitative approaches were applied, including descriptive aggregation of recovery, matrix effects, and precision across studies, as well as correlation analyses to explore relationships among performance parameters. **Results**: Twelve studies were included, spanning a range of typical and atypical antipsychotics and metabolites. Plasma and serum demonstrated the highest analytical reliability (recovery >90%, minimal matrix effects), while dried blood spots (DBSs), whole blood, and oral fluid showed greater variability. Clinically, UHPLC–MS/MS enabled more accurate dose adjustments and identification of non-adherence, outperforming immunoassays in sensitivity, specificity, and metabolite detection. Microsampling methods showed promise for outpatient and decentralized care but require further clinical validation. **Conclusions**: UHPLC–MS/MS remains the most robust and reliable method for TDM of antipsychotics, especially when quantification of active metabolites is required. While logistical barriers remain, technological advances may enhance feasibility and support broader integration into routine psychiatric care.

## 1. Introduction

Mental health disorders have a profound impact on patients, influencing nearly every aspect of their lives. These disorders frequently impose a substantial burden, extending beyond personal distress to include adverse long-term health outcomes, increased treatment challenges, and a diminished quality of life [1].

A significant challenge in the pharmacological management of mental disorders is the considerable variability in patient drug response, which requires careful optimization to maximize therapeutic efficacy while minimizing adverse effects [2]. TDM plays a critical role in optimizing the efficacy and safety of antipsychotic therapy, particularly given the narrow therapeutic windows, interindividual pharmacokinetic variability, and risk of adverse effects associated with these drugs [3,4].

Over the past two decades, Ultra-High Performance Liquid Chromatography–Tandem Mass Spectrometry (UHPLC-MS/MS) has transformed clinical laboratory practice by enhancing the monitoring of biomarkers, drugs, and metabolites with high speed and accuracy. Effective clinical monitoring of antipsychotics extends beyond measuring the parent compound, as drugs like risperidone, aripiprazole, and olanzapine are metabolized into active or toxic metabolites that influence both therapeutic efficacy and the risk of adverse effects. Given the influence of metabolic transformations, accurate quantification of both the parent drug and its active or toxic metabolites is essential. Metabolic variability affected by genetic polymorphisms, co-medications, and liver function can alter metabolite concentrations, thereby impacting treatment safety and outcomes [5,6,7,8].

Among analytical platforms available for TDM, UHPLC–MS/MS is widely regarded for its high sensitivity, specificity, and ability to simultaneously quantify multiple analytes. Compared to traditional HPLC or immunoassays, UHPLC–MS/MS offers superior resolution, faster run times, and greater ability to distinguish structurally similar compounds, including active metabolites that may influence clinical outcomes [9,10,11,12].

Advancements in microsampling (dried blood spots, oral fluid, and volumetric absorptive microsampling) have further expanded the applicability of UHPLC methods, allowing for less invasive and decentralized sample collection. In parallel, automated immunoassay platforms such as Alinity C have gained traction due to their ease of use and integration into routine clinical workflows. Despite good correlation in some studies, these platforms may lack the analytical specificity necessary to guide precise clinical decisions, particularly when metabolites are involved [13,14,15].

This systematic review examines the clinical applicability of UHPLC, with a focus on TDM of antipsychotic medications. Although UHPLC remains widely used, its role has not been systematically evaluated in recent years, despite significant advances in analytical technologies. The rapid evolution of liquid chromatography methods has produced a growing body of literature with inconsistent findings regarding UHPLC’s clinical utility [16,17,18,19]. This underscores the need for a structured synthesis of current evidence to assess whether UHPLC continues to serve as the method of choice in clinical evaluation [19,20,21,22,23,24].

In parallel with developments in analytical chemistry, psychiatry is undergoing a broader technological transformation. Emerging digital tools, wearable devices, and neurophysiological monitoring systems are reshaping how clinicians assess and manage mental health conditions. For example, Pergantis et al. (2025) reviewed assistive and emerging technologies for detecting stress and anxiety in autism and sensory processing disorders, underscoring how innovative methods are opening new possibilities for personalized care [25]. In this context, UHPLC–MS/MS should be viewed not only as a robust analytical platform but also as part of a broader movement towards precision and technology-enabled psychiatry.

While this method has been the reference one in many laboratories, the evolving analytical landscape, including increasing automation and cost-sensitive alternatives, raises a key question: can UHPLC–MS/MS, as the gold standard, still be considered a reliable and clinically valuable method for therapeutic drug monitoring of antipsychotics, offering sufficient advantages over other analytical techniques to justify its role in routine clinical practice [2,26,27]?

## 2. Materials and Methods

### 2.1. Protocol and Registration

This systematic review was conducted following PRISMA guidelines [28], and the protocol was prospectively registered in PROSPERO [29] under registration number CRD420251058505. The registered protocol outlines the objectives, eligibility criteria, search strategy, data extraction methods, and planned analyses to ensure methodological transparency and to minimize the risk of bias. No deviations from the registered protocol were made during the conduct of this review.

### 2.2. Eligibility Criteria

The inclusion criteria were as follows: (i) study designs such as methodological studies, analytical validation studies, or other non-randomized designs; (ii) peer-reviewed articles published in academic journals; (iii) studies evaluating UHPLC methods for measuring antipsychotic drug levels in clinical samples; (iv) adult participants, with or without a psychiatric diagnosis, receiving antipsychotic treatment; (v) publications in English; and (vi) studies using biological samples.

Exclusion criteria were: (i) reviews and meta-analyses; (ii) randomized controlled trials; (iii) studies not published in English; (iv) studies without human participants (in vitro experiments) or those involving pediatric populations; (v) studies analyzing medications other than antipsychotics; and (vi) studies evaluating only non-UHPLC methods or using non-biological samples.

### 2.3. Information Sources and Search Strategy

The databases analyzed were Web of Science, Scopus, and PubMed. A comprehensive search strategy was developed using a combination of controlled vocabulary and free-text keywords. The primary search terms included: “UHPLC” OR “Ultra-High-Performance Liquid Chromatography”, “Therapeutic Drug Monitoring” OR “TDM”, and “Antipsychotic”. These terms were combined using Boolean operators (AND, OR) to identify relevant studies. The search strategy was tailored to the specific indexing and search functionalities of each database (PubMed, Web of Science, and Scopus).

### 2.4. Study Selection

The screening process was conducted by two independent reviewers (B.M., M.C.), working in parallel and blinded to each other’s decisions. Any disagreements between the two reviewers that could not be resolved through discussion were referred to a third reviewer for adjudication (C.B.).

This review included articles published from 2013 to the present to capture recent advances in analytical techniques and clinical practices for measuring antipsychotic drugs. Limiting the timeframe to the last decade ensures the inclusion of up-to-date and clinically relevant data, reflecting improvements in bioanalytical methods while excluding outdated studies. All titles and abstracts were screened, and the full texts of potentially relevant studies were independently assessed by the same reviewers, all within the Rayyan platform [30]. A PRISMA flow diagram was used to document the study selection process, including the number of records identified, screened, excluded, and included in the final review. In cases where early online publication was cited, the print year was used for consistency, and the references are available in Table 1 and Table 2.

### 2.5. Data Extraction

Data were extracted from each included study using a standardized data extraction form, capturing information on study characteristics, analytical methods employed, types of biological samples analyzed, UHPLC parameters, drugs and metabolites investigated, and outcomes related to clinical utility or efficacy. Two reviewers independently performed the data extraction (B.M., M.C.), and any discrepancies were resolved through discussion or by consulting a third reviewer (C.B.).

### 2.6. Quality Assessment

Given the dominance of analytical method validation studies in the literature reviewed, standard risk of bias tools (ROBINS-I, QUADAS) were unsuitable. Consequently, a customized quality assessment checklist was developed, as outlined in Annex 1, drawing from international bioanalytical method validation guidelines (FDA, EMA, ICH Q2(R1)) [31,32,33].

This checklist evaluated six key domains. First, Study Design and Reporting examined the clarity of objectives, the relevance of sample matrices, and the sample preparation details. Second, Reference Standards and Controls assessed the use of certified materials and quality control measures. Third, Instrumentation and Method Parameters focused on detailed reporting of UHPLC systems and detection methods. Fourth, Analytical Performance covered calibration curves, linearity, sensitivity (LOD, LOQ), accuracy, precision, selectivity, and robustness. Fifth, Sample Analysis and Validation evaluated the use of real clinical samples, stability testing, and sufficient replication. Lastly, Data Reporting and Interpretation emphasized transparency in presenting results and discussing limitations. Each criterion was rated as “adequate”, “inadequate”, or “not reported” to systematically identify methodological strengths and weaknesses systematically, ultimately aiming to assess the rigor and clinical relevance of UHPLC validation studies.

Two reviewers independently assessed the quality of the studies (B.M., M.C.). Discrepancies between the reviewers were resolved through discussion or, when consensus was not reached, by consultation with a third reviewer. (C.B.)

### 2.7. Data Synthesis

Descriptive analyses were performed to summarize the included studies. A table was used to present the biological matrices investigated. Analytical method performance across antipsychotics was explored using scatter plots and bar charts. Correlations between matrix effects, recovery, and assay precision were examined through heat maps and scatter plots. Additionally, Pearson correlation analysis was conducted to explore relationships among key analytical parameters, matrix effects, recovery and precision, across the validation studies. The rationale for this analysis was to evaluate whether increased matrix effects were associated with reduced recovery or decreased precision, thereby assessing internal consistency and methodological robustness among the included datasets. In parallel, a qualitative assessment was carried out across all included studies, considering data such as study characteristics, analytical methods employed, types of biological samples analyzed, UHPLC parameters, the drugs and metabolites investigated, and outcomes related to clinical utility or efficacy.

For clarity, several technical terms are defined as follows: matrix effect refers to ion suppression or enhancement caused by co-extracted sample components; recovery denotes the proportion of analyte successfully extracted from the biological matrix; the coefficient of variation (CV) expresses analytical precision, calculated as (standard deviation/mean) × 100%.

In addition to this qualitative synthesis, non-traditional quantitative techniques tailored to analytical validation studies were employed. Owing to the heterogeneity of reported metrics, formal pooled meta-analysis was not feasible. Instead, descriptive aggregation was applied, averaging reported values across comparable compounds and matrices. Visual representations and correlation analyses were generated to explore relationships among performance parameters, with each study–drug–matrix combination treated as an independent datapoint. This structured approach enabled quantitative comparison beyond narrative reporting while preserving transparency regarding variability across studies.

## 3. Results

### 3.1. Study Selection

A total of 340 records were identified through database searches. After removing duplicates and screening titles and abstracts, 22 full-text articles were assessed for eligibility. Twelve studies met all inclusion criteria and were included in the final synthesis.

**Figure 1:**
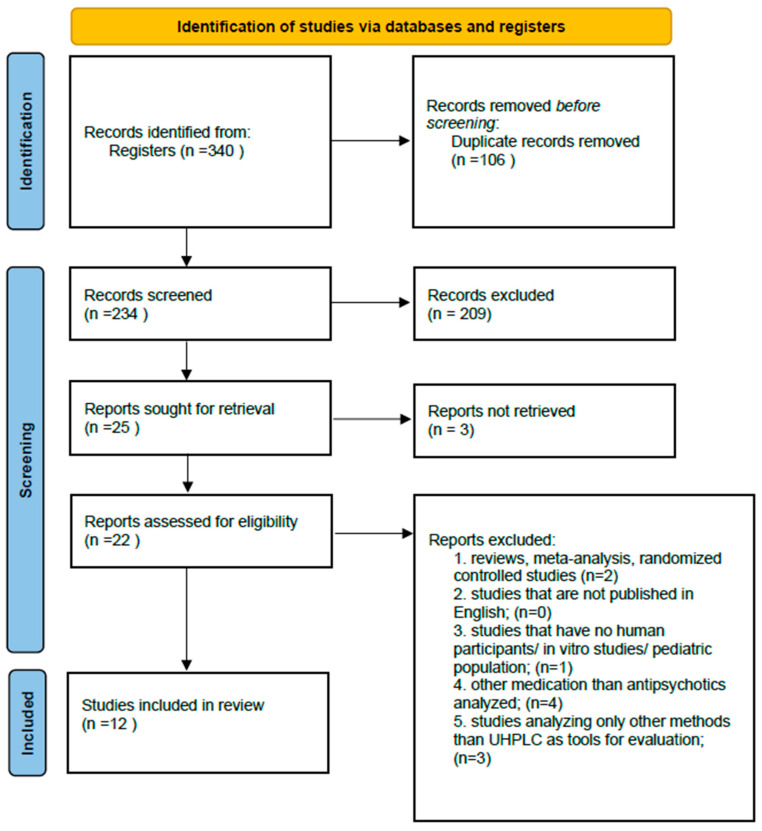
PRISMA Flow Diagram for the selection of studies included in the systematic review.

We created a PRISMA flow diagram to illustrate the study selection process transparently, ensuring methodological rigor and adherence to systematic review standards, as summarized in Figure 1 [34]. This diagram outlines the number of records identified, screened, assessed for eligibility and included in the final analysis, thereby enhancing the reproducibility and credibility of our research [34].

### 3.2. Characteristics of Included Studies

Twelve studies published between 2013 and 2024 were included, most of which were method development and validation studies involving UHPLC–MS/MS techniques [2,11,35,36,37,38,39,40,41,42,43,44]. Sample sizes ranged from 3 to 253 participants and study designs included both laboratory-based and clinical applications. While plasma and serum were the most commonly analyzed matrices, several studies also investigated alternative methods using DBS, WB, OF, and VAMS. The majority focused on second-generation antipsychotics such as risperidone, aripiprazole, and clozapine, often including their active metabolites. A few studies also compared UHPLC–MS/MS with automated immunoassay platforms (Alinity C). Table 1 provides a detailed overview of study characteristics, sample types and analytical techniques.

**Table 1:** Overview of Recent Studies on Antipsychotic Therapeutic Drug Monitoring Using Advanced Analytical Techniques and Alternative Sampling Methods.

	Author	Matrix	Antipsychotics Investigated	Participants	Analytical Method	Main Findings
1	Bernardo et al. (2022) [35]	DBS, Whole blood, Plasma	Aripiprazole, Clozapine, Paliperidone	31; Schizophrenia	UHPLC–MS/MS (Acquity UPLC + Waters triple quadrupole MS; ESI+, MRM)	DBS is a reliable, minimally invasive alternative to plasma/whole blood for antipsychotic TDM; good correlation with conventional matrices
2	Cruz et al. (2020) [37]	Human plasma	Chlorpromazine, Clozapine, Olanzapine, Quetiapine	11; Schizophrenia	UHPLC–MS/MS with RACNT microextraction; ESI+, MRM	RACNT-UHPLC–MS/MS method showed high sensitivity, precision and reusability for antipsychotic quantification in plasma; reduced matrix effects; suitable for routine TDM.
3	Millán-Santiago et al. (2023) [36]	VAMS and plasma	Cariprazine	3; Schizophrenia	UHPLC–MS/MS (Acquity UPLC + Xevo TQ-S; ESI+, MRM; validated for VAMS and plasma)	UHPLC–MS/MS with VAMS validated for cariprazine and metabolites; VAMS showed strong agreement with plasma; supports minimally invasive TDM.
4	Patteet et al. (2014) [39]	Seum	Amisulpride, Aripiprazole, Asenapine, Bromperidol, Clozapine, Haloperidol, Iloperidone, Levosulpiride, Lurasidone, Olanzapine, Paliperidone, Pipamperone, Quetiapine, Risperidone, Sertindole, Zuclopenthixol	12; analytical validation, not a clinical cohort	Acquity UPLC coupled to Xevo TQ-S triple quadrupole MS; ESI+; MRM; protein precipitation (high-throughput method)	High-throughput UHPLC–MS/MS validated for 16 antipsychotics + 8 metabolites in serum; accurate, precise, reproducible; suitable for routine TDM.
5	Patteet et al. (2014) [40]	Serum	Clozapine	171; diagnosis not specified (retrospective clozapine monitoring)	UHPLC–MS/MS (Acquity UPLC + Xevo TQ-S; ESI+, MRM; serum)	Retrospective evaluation of 171 patients; UHPLC–MS/MS reliably quantified clozapine/norclozapine; confirmed interindividual variability; supports clozapine TDM in routine practice.
6	Patteet et al. (2015) [41]	DBS, Plasma	Amisulpride, Aripiprazole, Asenapine, Bromperidol, Clozapine, Haloperidol, Iloperidone, Lurasidone, (Levo)sulpiride, Olanzapine, Paliperidone, Pipamperone, Quetiapine, Risperidone, Zuclopenthixol	171; (same clinical cohort as their serum studies)	UHPLC-MS/MS APs Metabolites Quantitative analysis TDM Bioanalysis Plasma analysis	DBS validated against plasma for 15 antipsychotics + 7 metabolites; good agreement; minimally invasive alternative for TDM.
7	Patteet et al. (2016) [42]	Serum	Aripiprazole, Haloperidol, Risperidone, Paliperidone, Zuclopenthixol	82 psychiatric patients (serum samples analyzed; cohort characterized for CYP2D6 genotype)	UHPLC–MS/MS (Acquity UPLC + Xevo TQ-S; ESI+, MRM; serum, CYP2D6 impact study)	CYP2D6 genotype and co-medication significantly affected serum levels of several antipsychotics; highlights importance of pharmacogenetics in TDM
8	Patteet et al. (2016) [43]	Oral fluid	Amisulpride, Aripiprazole, Clozapine, Haloperidol, Olanzapine, Quetiapine, Risperidone, Sertindole, Ziprasidone	82 psychiatric patients (matched with serum samples from the same cohort)	UHPLC–MS/MS (Acquity UPLC + Xevo TQ-S; ESI+, MRM; validated for oral fluid)	UHPLC–MS/MS validated for 9 antipsychotics in oral fluid; variable correlation with serum; oral fluid less reliable than plasma/DBS for TDM.
9	Pouliopoulos et al. (2018) [44]	Serum, Postmortem blood	Amisulpride, Aripiprazole, Asenapine, Chlorpromazine, Clozapine, Haloperidol, Levomepromazine, Olanzapine, Paliperidone, Perphenazine, Quetiapine, Risperidone, Sulpiride, Ziprasidone, Zuclopenthixol	Method validation study; included postmortem samples and small-volume serum samples	UHPLC–MS/MS (Shimadzu Nexera X2 UHPLC system coupled to a Shimadzu 8040 triple quadrupole MS; ESI+, MRM), validated for serum and postmortem blood.	UHPLC–MS/MS validated for 15 antipsychotics in small serum/postmortem volumes; accurate and robust; applicable in clinical TDM and forensic toxicology
10	Toja-Camba et al. (2024) [2]	Plasma	Risperidone, Paliperidone	115; 92 on risperidone, 23 on paliperidone	UHPLC–MS/MS (Acquity UPLC + Xevo TQ-S; ESI+, MRM; plasma; compared with Alinity C immunoassay)	UHPLC–MS/MS and Alinity C showed overall agreement for risperidone/paliperidone; immunoassay consistently overestimated levels; UHPLC–MS/MS remains reference method.
11	Toja-Camba et al. (2024) [11]	Plasma	Aripiprazole	86 patients treated with aripiprazole	UHPLC–MS/MS (Acquity UPLC + Xevo TQ-S; ESI+, MRM; plasma; compared with Alinity C immunoassay)	“UHPLC–MS/MS accurately quantified aripiprazole and metabolite; immunoassay overestimated levels; supports UHPLC–MS/MS as reference method for precision psychiatry.”
12	Wang et al. (2017) [38]	Plasma	Aripiprazole, Amisulpride, Olanzapine, Paliperidone, Ziprasidone	253 patients with schizophrenia	UHPLC–MS/MS (Ekspert ultraLC 100-XL + QTRAP 5500; ESI+, MRM; plasma)	Validated UHPLC–MS/MS for 5 antipsychotics in plasma; accurate and reproducible; suitable for routine TDM in schizophrenia.

(DBS—Dried Blood Spot, VAMS—Volumetric Absorptive Microsampling, UHPLC–MS/MS—Ultra-High Performance Liquid Chromatography–Tandem Mass Spectrometry, UPLC-MS/MS—Ultra Performance Liquid Chromatography-Tandem Mass Spectrometry, MRM—Multiple Reaction Monitoring, RACNTs—Raw Amino-Functionalized Carbon Nanotubes, TDM—Therapeutic Drug Monitoring, AGNP—Arbeitsgemeinschaft für Neuropsychopharmakologie und Pharmakopsychiatrie, CLO—Clozapine, NORCLO—Norclozapine, ARI—Aripiprazole, HAL—Haloperidol, RIS—Risperidone, ZUC—Zuclopenthixol, OF—Oral Fluid).

### 3.3. Quality Assessment

A custom quality assessment checklist was developed based on international bioanalytical validation guidelines (FDA, EMA, ICH Q2(R1)) to evaluate study rigor across six domains [31,32,33]. Overall, most included studies demonstrated adequate reporting in instrumentation, calibration procedures and precision parameters. Several studies lacked detailed descriptions of stability testing and matrix-specific validation, particularly for alternative sampling methods such as DBS and OF. The overall methodological quality of the studies was deemed acceptable, justifying their inclusion in the synthesis. A domain-by-domain summary is available in Annex 1. Having characterized the included studies and evaluated their methodological quality, the following sections summarize the quantitative performance of UHPLC-based assays across biological matrices, parent compounds, and metabolites.

### 3.4. Quantitative Assessment

Quantitative analyses were performed to assess the analytical efficiency and reproducibility of UHPLC-based methods across studies, focusing on recovery, matrix effects, and precision parameters for both parent antipsychotic drugs and their metabolites.

Of the 12 included studies, 9 provided sufficient data for performance evaluation and 8 were eligible for quantitative synthesis due to method duplication in one study [39,40]. The quantitative analysis focused on the 8 studies specifically addressing method validation focusing on drug parents and metabolites, while data from the remaining studies were evaluated qualitatively in other sections to provide a comprehensive overview [35,36,37,38,39,43,44].

#### 3.4.1. Biological Matrices Investigated in Included Studies

Across the 12 studies included, seven different biological matrices were used for UHPLC-based antipsychotic analysis [2,11,35,36,37,38,39,40,41,42,43,44]. Four matrices were assessed in more than one study: serum, DBS, plasma and WB, each investigated twice [35,37,38,39,41,44]. Less commonly used matrices included plasma processed by SPE, OF and VAMS, each assessed in a single study, as seen in Table 2.

**Table 2:** Overview of Sample Matrices Utilized in UHPLC-Based Clinical Studies.

Matrix	Study	Number of Studies
**Serum**	Patteet et al., 2014 [39], Pouliopoulos et al., 2018 [44]	2
**DBS**	Bernardo et al., 2022 [35], Patteet et al., 2014 [39,40]	2
**Plasma**	Cruz et al., 2023 [37], Wang et al., 2017 [38]	2
**WB**	Bernardo et al., 2022 [35], Millán-Santiago et al., 2023 [36]	2
**SPE**	Pouliopoulos et al., 2018 [44]	1
** OF**	Patteet et al., 2016 [42,43]	1
**VAMS**	Millán-Santiago et al., 2023 [36]	1

(DBS—Dried Blood Spot, WB—Whole Blood, SPE—Solid Phase Extraction, OF—Oral Fluid, VAMS—Volumetric Absorptive Microsampling).

#### 3.4.2. Analytical Method Performance Across Antipsychotics

Reported recovery, matrix effect and CV values were extracted as means or ranges when available, then averaged per compound across studies.

**Figure 2:**
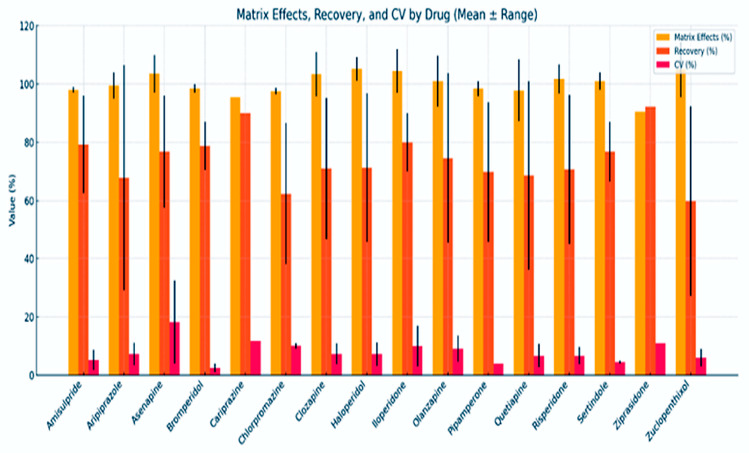
Analytical Performance Parameters (Matrix Effects, Recovery and CV) for UHPLC Quantification of Antipsychotic Drugs. (CV—Coefficient of Variation, ME—Matrix Effects, REC—Recovery). Plasma and serum provide the most consistent performance, confirming their role as preferred matrices for clinical TDM.

Analytical performance varied across the evaluated antipsychotic drugs, as seen in Figure 2. Matrix effects were generally controlled, with most drugs showing mean values near 100%. Matrix effects express ion suppression or enhancement relative to a pure standard solution. Values below 100% indicate signal suppression (e.g., 95% corresponds to a 5% loss in signal), whereas values above 100% indicate signal enhancement due to matrix-related interference. Exceptions included Asenapine and Iloperidone, which displayed broader variability. Bars show the mean values of matrix effects, recovery and CV per drug, with error bars representing the reported range across studies. No inferential statistics were applied, as the purpose was descriptive comparison of analytical performance across compounds.

Recovery rates showed greater variability between drugs: Amisulpride, Chlorpromazine and Ziprasidone showed high recovery (>90%), while Asenapine, Clozapine and Sertindole had moderate to low recovery with wider ranges. Precision was acceptable across most drugs (mean CV < 10%), though Asenapine and Cariprazine exhibited greater variability.

#### 3.4.3. Analytical Method Performance Across Antipsychotic Metabolites

For each metabolite, recovery, matrix effect and CV values were extracted from available studies and averaged; ranges were retained when reported.

**Figure 3:**
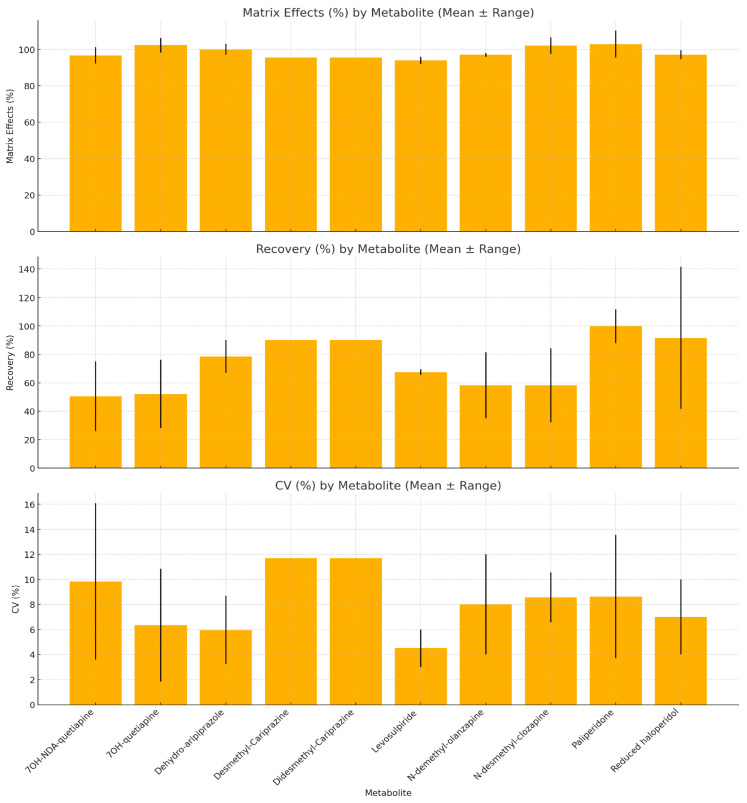
Analytical Performance of UHPLC for Metabolite Quantification: Matrix Effects, Recovery and Coefficient of Variation (Mean ± Range) (CV—Coefficient of Variation, ME—Matrix Effects, REC—Recovery). UHPLC reliably detects clinically relevant metabolites, supporting its use in dose optimization and adherence monitoring.

With growing interest in monitoring antipsychotic metabolites for treatment adherence, several studies evaluated their analytical performance using UHPLC, with detailed data presented in Figure 3. For each metabolite, mean values of matrix effects, recovery and CV were calculated across studies, with ranges shown as error bars.

Recovery varied by compound: Cariprazine metabolites showed consistently high recovery (≈90%), whereas hydroxylated quetiapine metabolites had lower, more variable recovery (≈50%). Matrix effects were generally stable (95.5–102.3%). 7OH-quetiapine had the highest mean matrix effect (>102%), while Desmethyl- and Didesmethyl-Cariprazine displayed the lowest, with minimal variability.

Precision was acceptable overall. Dehydro-aripiprazole showed the lowest CV (≈6%), while Cariprazine metabolites averaged 11.7% and 7OH-NDA-quetiapine reached a CV of up to 16.5%.

#### 3.4.4. Analytical Performance Relationships Across Matrices

Correlation analyses were conducted by aligning validation parameters across eight studies, treating each study–drug–matrix combination as an independent datapoint.

This statistical approach aimed to identify whether variability in matrix effects influenced recovery or analytical precision and to determine the internal consistency of UHPLC-based performance metrics across different biological matrices.

**Figure 4:**
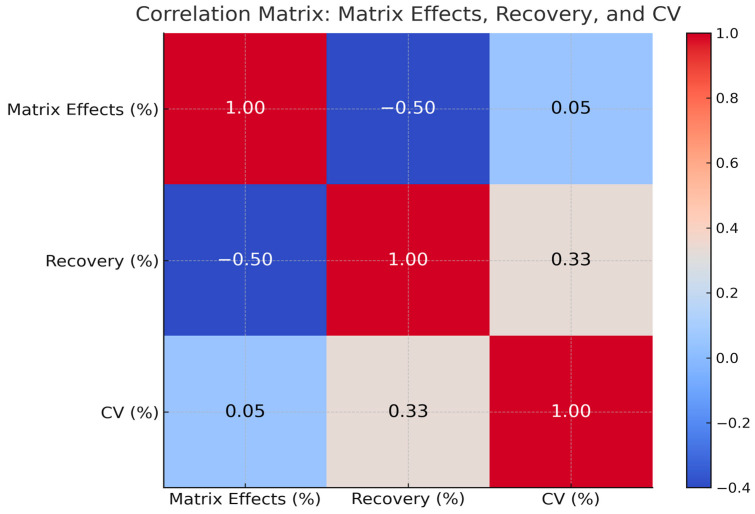
Pearson Correlation Coefficients Between Analytical Performance Parameters in UHPLC-Based Studies (CV—Coefficient of Variation, ME—Matrix Effects, REC—Recovery). Correlations confirm that recovery and precision remain within clinically acceptable ranges, reinforcing the robustness of UHPLC assays.

Correlation analysis assessed the relationships between matrix effects, recovery, and precision across included UHPLC studies (Figure 4). The strength and direction of these associations were evaluated using Pearson’s correlation coefficient (r), where values closer to ±1 indicate stronger linear relationships. A moderate inverse correlation was found between matrix effects and recovery (r = −0.50). Matrix effects and CV% showed minimal correlation (r = 0.05). A weak positive correlation was observed between recovery and CV% (r = 0.33).

**Figure 5:**
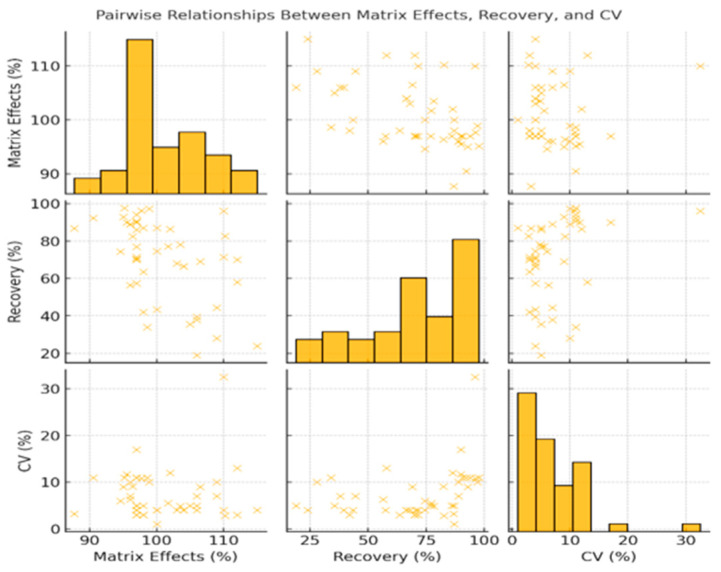
Pairwise Analysis of Matrix Effects, Recovery and Coefficient of Variation in UHPLC-Based Clinical Assays (CV—Coefficient of Variation, ME—Matrix Effects, REC—Recovery). Most assays achieve recovery above 75% and precision below 10%, meeting regulatory standards for clinical implementation.

Figure 5 presents a pairwise scatterplot of these parameters. Matrix effects ranged from 95% to 105%. Recovery ranged from 20% to 100%, with most values between 75 and 95%. CV% values were primarily below 10%, with a few above 20%.

Pairwise scatterplots with marginal histograms were generated to visualize the distribution of each variable and assess potential linear relationships between matrix effects, recovery and CV.

#### 3.4.5. Recovery in Relation to Matrix Effects for Antipsychotics

When multiple studies reported on the same drug in the same matrix, results were averaged; single-study data were presented as reported.

Due to the heterogeneity of study designs, target compounds and validation procedures, a formal inferential comparison across biological matrices could not be performed. Instead, a descriptive cross-matrix analysis was carried out. This comparison indicated that plasma and serum generally achieved higher mean recovery (90–100%) and lower inter-assay variability (CV < 10%), whereas DBS and oral fluid samples showed reduced extraction efficiency and slightly greater precision variability. These trends suggest that matrix composition substantially influences both recovery and analytical robustness in UHPLC–MS/MS assays.

Analytical recovery and matrix effects were compared across six biological matrices using averaged values from eight included studies. Only antipsychotic-specific data from Pouliopoulos et al. were considered [44].

**Figure 6:**
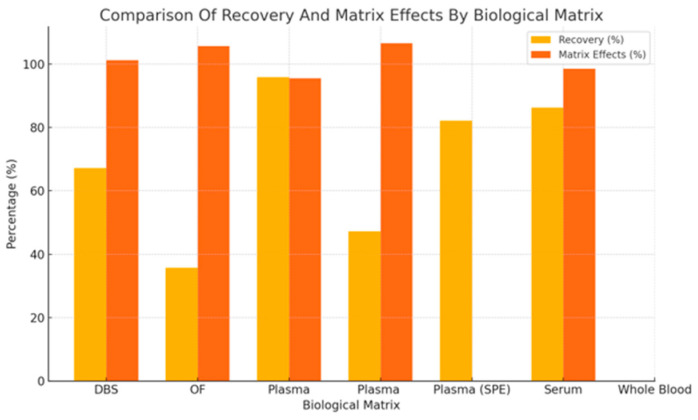
Comparison of Recovery and Matrix Effects Across Different Biological Matrices in UHPLC-Based Assays (DBS—Dried Blood Spot, OF—Oral Fluid, SPE—Solid Phase Extraction). Plasma remains the gold standard, while DBS offers moderate reliability with major advantages in accessibility for patients.

As summarized in Figure 6, plasma showed the highest mean recovery (95.8%) with moderate matrix effects (95.6%), serum had recovery of 86.2% and matrix effects of 98.5%, and SPE yielded 82.2% recovery; matrix effect data were inconsistently reported, DBS showed lower recovery (67.2%) with matrix effects at 101.3%, and OF had the lowest recovery (35.8%) and the highest matrix effects (105.6%). WB was underrepresented in the dataset, with no available values for recovery or matrix effects.

#### 3.4.6. Recovery in Relation to Matrix Effects for Metabolites of Antipsychotics

Available metabolite data were aggregated by matrix, with averages calculated where multiple studies reported on the same compound; gaps were indicated where data were not available.

**Figure 7:**
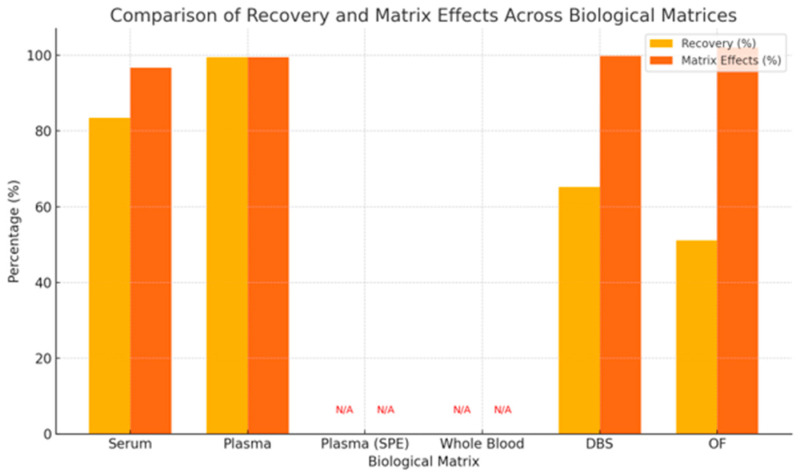
Recovery and Matrix Effects Across Biological Matrices: Data Availability and Performance Variation in UHPLC Assays (DBS—Dried Blood Spot, OF—Oral Fluid, SPE—Solid Phase Extraction). Plasma and serum provide the highest reliability, while DBS and OF, though less robust, may improve adherence through minimally invasive sampling.

Average recovery and matrix effects for antipsychotic metabolites were compared across six biological matrices based on available data, as seen in Figure 7.

Plasma showed the highest recovery (99.5%) and matrix effects (99.5%), and serum exhibited recovery of 83.5% with matrix effects of 96.75%, DBS and OF showed lower recoveries (65.17% and 51.08%, respectively), while matrix effects remained near 100%. Data for SPE and WB were not available, indicated by red “N/A” markers in the figure, reflecting gaps in reporting or underrepresentation of these matrices in validation studies.

#### 3.4.7. Impact of Recovery on Method Precision (CV/Bias) on Parent Drugs and Metabolites

Recovery and CV/bias values were extracted from validation datasets, with each drug–matrix pair considered as a distinct datapoint to assess the relationship between recovery and assay precision.

**Figure 8:**
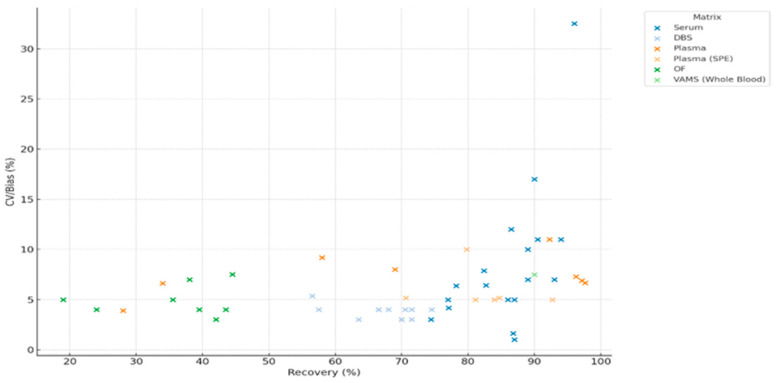
Relationship Between Recovery and CV/Bias Across Biological Matrices in UHPLC-Based Clinical Assays (DBS—Dried Blood Spot, OF—Oral Fluid, SPE—Solid Phase Extraction, VAMS—Volumetric Absorptive Microsampling).

Recovery (%) and precision/bias (%) data were extracted from eight eligible studies reporting analytical validation of antipsychotic drug measurements across different biological matrices [2,11,35,36,37,38,39,40,41,42,43,44]. For the study of Pouliopoulos et al. [44], only data relating to antipsychotic compounds were included. A scatter plot was generated to display the relationship between recovery and assay precision across drug–matrix pairs (Figure 8).

**Figure 9:**
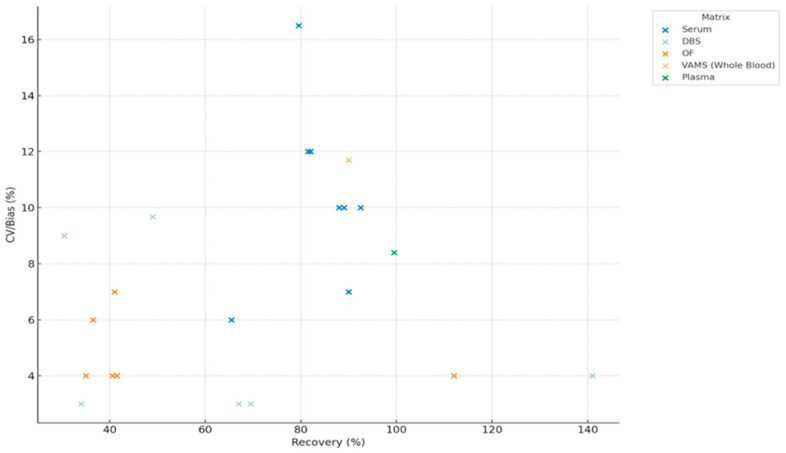
Distribution of Recovery and CV/Bias Across Biological Matrices in UHPLC Assays (DBS—Dried Blood Spot, OF—Oral Fluid, VAMS—Volumetric Absorptive Microsampling, WB—Whole Blood, SPE—Solid Phase Extraction).

A total of 16 antipsychotic drug–matrix combinations were identified across six biological matrices. Recovery (%) was plotted against a combined CV/Bias (%) measure for each drug–matrix pairing (Figure 9).

For metabolites, average analytical recovery and matrix effects were extracted for five biological matrices: serum, plasma, DBS, OF, and WB/VAMS (plasma processed by SPE was not reported for metabolites). The metabolite analysis included nine compounds related to antipsychotics. N-desmethyl-clozapine, 7-hydroxy-quetiapine and paliperidone were most frequently reported, particularly in plasma, serum and DBS.

### 3.5. Qualitative Assessment

In addition to the quantitative synthesis, a qualitative analysis was conducted to contextualize the analytical findings in terms of clinical applicability, study populations, and sample preparation methods.

A qualitative assessment was conducted for all included studies, examining study characteristics, analytical methods, biological samples, UHPLC parameters, investigated drugs and metabolites and clinical relevance.

#### 3.5.1. Study Populations and Antipsychotic Medications

Among the included studies, most participants were diagnosed with schizophrenia. Four studies did not specify a diagnosis; three involved patients with bipolar disorder, two with schizoaffective disorder and one included healthy volunteers. The antipsychotics analyzed were primarily atypical agents, including aripiprazole, clozapine, risperidone, cariprazine, quetiapine, olanzapine and ziprasidone. Some studies also evaluated typical antipsychotics such as haloperidol and chlorpromazine [2,11,35,36,37,38,39,40,41,42,43,44]. Most studies quantified both parent compounds and their pharmacologically active metabolites. Commonly reported metabolites included dehydro-aripiprazole (aripiprazole), N-desmethylclozapine (clozapine), paliperidone and 9-hydroxyrisperidone (risperidone), desmethyl- and didesmethyl-cariprazine (cariprazine), 7-hydroxyquetiapine and 7-hydroxy-N-desalkyl-quetiapine (quetiapine), N-desmethyl-olanzapine (olanzapine) and reduced haloperidol (haloperidol) [38,39].

Metabolite data were less frequently reported for other compounds such as amisulpride, asenapine and iloperidone.

#### 3.5.2. Comparative Assessment of UHPLC-MS/MS and Alinity C Methods for Antipsychotic Quantification

Two studies were identified that performed direct comparisons of UHPLC-MS/MS and Alinity C methods for the quantification of antipsychotic drugs in plasma. The first study [2] focused on risperidone and paliperidone, while the second [11] investigated aripiprazole and dehydroaripiprazole. A more detailed analysis of these studies is provided in Table A2 (Appendix B).

For risperidone and paliperidone, UHPLC-MS/MS (Waters, Milford, MA, USA) was performed using a Xevo TQDR triple quadrupole mass spectrometer with risperidone-d4 as the internal standard. The method had a linear calibration range of 1–200 ng/mL (R^2^ = 0.99) and limits of detection and quantification of 0.5 ng/mL and 1 ng/mL, respectively. In comparison, the Alinity C (Abbott, Abbott Park, IL, USA) method used a competitive immunoassay with photometric detection at 604 nm and a quantification range of 16–120 ng/mL. A total of 115 plasma samples were analyzed using both methods, with the results stratified into infra-therapeutic, therapeutic and supra-therapeutic categories. Additional analyses examined risperidone-to-paliperidone ratios.

In the aripiprazole study, UHPLC-MS/MS again used the Xevo TQDR instrument, with aripiprazole-d8 as the internal standard. The calibration range extended from 25 to 1000 ng/mL (R^2^ = 0.998), and limits of detection and quantification were 10 ng/mL and 25 ng/mL, respectively. The Alinity C method employed the same detection principle and kit, offering a quantification range of 45–1000 ng/mL. Sixty of the 86 total plasma samples were analyzed with both methods. The data were stratified by therapeutic ranges and aripiprazole-to-dehydroaripiprazole ratios were also examined. Additionally, a subset of 26 samples was reanalyzed after six months to assess assay reproducibility.

Both studies documented instrument parameters, calibration ranges, internal standards and performance characteristics. These data allowed for a direct comparison of the analytical performance and clinical utility of UHPLC-MS/MS versus immunoassay methods in therapeutic drug monitoring of antipsychotics.

#### 3.5.3. UHPLC-MS/MS in Personalized Therapeutic Drug Monitoring

Among the twelve studies included [2,11,35,36,37,38,39,40,41,42,43,44], one [42] applied UHPLC-MS/MS not only for quantifying antipsychotic serum levels but also to assess variability linked to patient metabolism.

In this study, 82 psychiatric patients were genotyped and classified into metabolizer categories: poor (14.6%), intermediate (6.1%), extensive-slow (46.3%), extensive-fast (25.6%), ultra-rapid (6.1%) and one mixed phenotype. CYP2D6 activity scores were adjusted for potential phenoconversion due to co-medication.

Serum concentrations of aripiprazole, haloperidol, risperidone and zuclopenthixol were significantly influenced by CYP2D6 metabolic status, whereas paliperidone levels remained unaffected.

#### 3.5.4. Assessment of UHPLC Sample Preparation Techniques for Routine Clinical Implementation and Storage Conditions

Sample preparation methods varied across studies, with LLE being the most common technique, particularly for serum samples. Studies by Patteet et al. [39,40,41] and Pouliopoulos et al. [44] applied LLE with methyl tert-butyl ether (MTBE), followed by evaporation and reconstitution in acetonitrile, supporting routine TDM. Protein precipitation using acetonitrile or methanol was also widely employed, especially in plasma-based workflows (Toja-Campa et al., Wang et al., Cruz et al.), offering a quick and clinically suitable approach. More novel techniques such as MEPS with RACNT and VAMS were reported in fewer studies, with partial suitability due to ongoing implementation challenges.

In terms of matrices, plasma was the most frequently analyzed biological sample (n = 5), followed by serum, WB, DBS and OF. Storage conditions ranged from room temperature to −80 °C, although several studies did not fully report temperature parameters. Plasma and serum samples were typically stored at −20 °C or −80 °C, while DBS and VAMS samples were stored at room temperature.

A detailed overview of sample preparation protocols and clinical applicability is provided in Table 3.

## 4. Discussion

This systematic review assessed the clinical application of UHPLC, which remains widely used due to its high sensitivity, specificity, and throughput.

### 4.1. Biological Matrices and Analytical Variability: A Multidimensional Challenge

This review highlights the versatility of UHPLC and UHPLC-MS/MS across diverse biological matrices, including plasma, serum, WB, DBS and VAMS for TDM of antipsychotics.

Plasma remains the preferred matrix due to its high analytical reliability, minimal matrix effects and widespread clinical acceptance. Although serum demonstrates comparable performance, subtle concentration biases emphasize the need for matrix-specific validation protocols [38,45,46]. The choice of matrix not only affects analytical accuracy but also impacts patient comfort, sample collection feasibility and turnaround times, which are critical considerations in clinical settings [47].

Antipsychotic medications are primarily prescribed for patients with schizophrenia and related psychotic disorders, populations that often face challenges with medication adherence and frequent clinical visits. The adoption of minimally invasive sampling methods such as DBS and VAMS can significantly improve patient comfort and accessibility to therapeutic monitoring, facilitating more frequent and convenient assessments. This can lead to timely dose adjustments tailored to individual pharmacokinetic profiles, ultimately enhancing treatment efficacy and minimizing adverse effects [35,36,40,48,49]. While plasma remains the reference matrix for antipsychotic drug monitoring, patient-centered factors must also be considered. Many psychiatric patients experience significant distress with repeated venipuncture, which can limit the feasibility of frequent monitoring. Minimally invasive sampling approaches such as DBS and VAMS offer practical advantages by reducing discomfort, simplifying collection and enabling decentralized or even home-based sampling. These approaches may increase patient willingness to engage in therapeutic drug monitoring, thereby supporting adherence and continuity of care. Although analytical performance for these matrices can be more variable compared to plasma, their patient-centered advantages highlight the importance of further optimization and validation to facilitate wider clinical adoption [50,51]. WB analysis offers practical advantages but requires optimized methods to address matrix complexities [35,52]. Integrating these flexible sampling strategies into routine care holds promise for supporting more patient-centered management of complex psychiatric conditions.

Apart from analytical considerations, patient-related factors such as pharmacogenetics and concomitant medications also influence serum drug concentrations. Patteet et al. (2016) [42] demonstrated that CYP2D6 genotype and co-medication significantly affected antipsychotic serum levels, highlighting the importance of integrating pharmacogenetic information into TDM.

The consistent detection of both parent compounds and active metabolites across studies underscores UHPLC’s capability to provide comprehensive pharmacokinetic profiles essential for personalized therapy. Frequent quantification of metabolites, such as dehydro-aripiprazole, norclozapine and reduced haloperidol, reflects their clinical significance in efficacy and safety monitoring [11,39,40]. Variability in metabolite reporting for some antipsychotics may indicate either analytical constraints or limited metabolic transformation, highlighting areas for future research. Overall, this analysis supports UHPLC’s adaptability to evolving clinical workflows and its critical role in advancing patient-centered therapeutic monitoring of antipsychotic medications, particularly for vulnerable populations requiring individualized care.

### 4.2. Analytical Performance Across Drugs and Metabolites: A Case for UHPLC-MS/MS

The evaluation of UHPLC–MS/MS methods across studies demonstrated consistently reliable analytical performance for antipsychotic drug quantification. However, as no direct statistical comparisons with alternative analytical techniques were performed, this conclusion should be interpreted as evidence of internal consistency rather than superiority over other methods [31,32]. Slight ion enhancement or suppression occurred for select compounds, but no analyte interferences required protocol modifications [44,45]. Recovery rates were robust (75–100%), markedly exceeding legacy HPLC-UV approaches, especially in plasma and DBS, and precision was excellent, with intra- and inter-assay CVs usually below 10% [15,45,46]. These findings reinforce UHPLC-MS/MS’s reliability in clinical pharmacology.

Across validation studies, *accuracy* was generally reported through recovery experiments, with mean values ranging from 75% to 100%, depending on the compound and biological matrix. Plasma-based methods consistently achieved recoveries above 90%, while alternative matrices such as DBS and oral fluid showed greater variability, reflecting matrix-dependent extraction efficiency. *Precision* was evaluated through intra- and inter-day analyses and remained within the 15% CV limit recommended by FDA, EMA, and ICH Q2(R1) guidelines in nearly all cases, confirming satisfactory assay reproducibility. *Specificity* was verified by analyzing blank and matrix-spiked samples to rule out endogenous or co-medication interferences, particularly relevant in psychiatric cohorts where polypharmacy is common. *Limits of detection (LOD)* and *quantification (LOQ)* typically ranged from 0.5 to 10 ng/mL and 1 to 25 ng/mL, respectively, which is sufficient to cover both therapeutic and subtherapeutic drug concentrations. *Robustness* was assessed in several studies by introducing minor, deliberate variations in chromatographic conditions (e.g., column temperature, mobile phase composition, flow rate), confirming that analytical performance remained stable within regulatory expectations. Collectively, these findings indicate that the included validation studies adhered to international bioanalytical standards and provide a reliable foundation for clinical implementation of UHPLC–MS/MS in therapeutic drug monitoring [31,33].

Metabolite quantification yielded variable recoveries due to compound-specific factors like lipophilicity and protein binding, although matrix effects remained controlled (85–105%) [44,45]. Precision remained within acceptable bioanalytical limits (<15%), particularly when leveraging matrix-matched calibration and isotope-labeled standards [43]. The capability of UHPLC-MS/MS to simultaneously quantify parent drugs and active metabolites, essential for profiling drug–metabolite pairs such as clozapine/norclozapine and risperidone/9-hydroxyrisperidone, further adds to its clinical utility [44,53].

Significantly, UHPLC-MS/MS has been shown to identify misclassifications made by immunoassays in real clinical scenarios. In one comparison of UHPLC-MS/MS versus the Alinity C immunoassay system for risperidone and paliperidone, UHPLC revealed that immunoassay-based results overestimated concentrations by nearly 1 ng/mL in 16 % of patient samples, with lower agreement (κ = 0.63) for paliperidone measurements, discrepancies that could influence dosing decisions in patients near therapeutic thresholds [2]. Additionally, case reports have shown that risperidone and its active metabolite, 9-hydroxyrisperidone, can produce false-positive results on fentanyl immunoassay screens when tested in urine. This cross-reactivity has the potential to lead to misinterpretation in psychiatric or forensic contexts, particularly when confirmatory testing is not performed [54]. These findings highlight the clinical importance of UHPLC-MS/MS in correcting erroneous results that could otherwise compromise patient safety or treatment decisions.

Overall, UHPLC-MS/MS surpasses immunoassay-based tools, like Alinity C or rapid drug screens, in sensitivity, specificity, dynamic range and ability to differentiate between antipsychotics and interfering substances [53,55,56]. This enhanced analytical clarity supports more accurate therapeutic drug monitoring, dose adjustments and management of adverse events, especially in patients with chronic psychosis who often face adherence challenges or borderline therapeutic concentrations [57]. UHPLC-MS/MS consequently stands out as a clinically valuable platform that can prevent misclassification, support individualized dosing and ultimately improve patient outcomes in psychiatric care.

In addition to its analytical strengths, the clinical relevance of UHPLC–MS/MS is closely tied to patient outcomes. TDM offers a practical tool for optimizing antipsychotic treatment in real-world settings, where pharmacokinetic variability and narrow therapeutic ranges often complicate management. Reliable measurement of drug levels allows clinicians to make well-informed dose adjustments in patients at risk of underexposure or toxicity. Moreover, the ability to detect active metabolites adds an additional layer of precision, ensuring that treatment decisions are based on a comprehensive pharmacological profile. Importantly, TDM has also been shown to support the early identification of non-adherence, a leading cause of relapse and rehospitalization in psychiatric care. By providing objective data to guide interventions, UHPLC–MS/MS contributes to improved adherence, reduced relapse rates, and overall better clinical outcomes. These advantages underscore its role not only as a robust analytical platform but also as a tool that can meaningfully influence long-term treatment trajectories in psychiatry [5].

### 4.3. Recovery, Matrix Effects, and Precision: Navigating Analytical Trade-Offs

Recovery and matrix effects are key parameters in UHPLC-MS/MS validation, directly influencing assay accuracy and clinical reliability. In this review, plasma consistently showed the highest recovery (99.5%) and minimal matrix effects (99.5%), reaffirming its role as the gold standard for antipsychotic quantification [46,58]. Serum also performed well (recovery 87%, matrix effects 98%) but tends to show a slight positive bias versus plasma due to clot-related cellular release, requiring matrix-specific validation [15,39].

Alternative matrices such as DBS and OF present practical advantages, particularly for remote or outpatient settings, but come with trade-offs. DBS had moderate recovery (67%) and acceptable matrix effects (101%) but suffers from hematocrit-related variability (6.54). OF showed poor recovery (35%) and high matrix effects (107%), limiting its utility for quantitative applications without further optimization [43,59,60]. VAMS has shown promise in balancing convenience with analytical performance and may enhance access to TDM in decentralized care models [61].

WB was largely absent from the dataset, reflecting its current underuse due to analytical complexity and limited standardization. Where tested, recovery and matrix effects were highly variable, depending on drug properties and extraction protocols [6]. Although WB offers logistical benefits, it currently lacks the robustness and clinical comparability of plasma-based methods.

While UHPLC-MS/MS requires a higher upfront investment compared to traditional methods like immunoassays or HPLC-UV, it offers significantly better specificity, sensitivity and throughput. These advantages translate to lower long-term costs per data point, particularly when quantifying multiple drugs or metabolites in a single run [53,55,62]. The improved accuracy may reduce the need for repeat testing and minimize the clinical risks associated with misclassification or dosing errors, ultimately supporting more cost-effective and individualized patient care. This confirms as well that UHPLC-MS/MS is clinically and economically valuable, especially for guiding TDM in psychiatric populations, where adherence issues and narrow therapeutic windows require precise and reproducible monitoring [53,56].

### 4.4. Toward Clinical Integration: The Role of UHPLC in Personalized Medicine

The integration of UHPLC-MS/MS into TDM for antipsychotics has established a benchmark for analytical accuracy, sensitivity and specificity, particularly for drugs with narrow therapeutic windows such as clozapine, risperidone and aripiprazole [15]. Multiplexed UHPLC-MS/MS methods enable simultaneous quantification of multiple antipsychotics and metabolites with high throughput and minimal matrix effects, facilitating comprehensive and efficient patient monitoring. Our review confirms UHPLC-MS/MS as a widely validated approach for TDM across plasma, saliva and DBS samples. DBS methods demonstrate moderate correlation with venous plasma concentrations, highlighting their clinical utility and minimally invasive nature [35]. While UHPLC-MS/MS offers exceptional analytical capabilities, its adoption requires substantial initial investment in equipment and ongoing maintenance, as well as skilled personnel trained in complex sample preparation and data interpretation. Additionally, variability in assay standardization and validation across laboratories presents obstacles to consistent implementation and comparability of results in clinical practice [63]. Overcoming these challenges through method harmonization, increased automation and the development of more cost-effective workflows is essential to maximize the clinical advantages and precision of UHPLC-MS/MS in personalized psychiatric treatment.

While alternative approaches such as immunoassays or high-resolution mass spectrometry (HRMS) offer faster workflows, they generally lack the sensitivity and reproducibility of UHPLC-MS/MS, which is critical for dose optimization and adherence verification in psychiatric care [11]. Clear information on sample preparation is vital for clinicians and labs to ensure accurate and consistent drug measurements. Reliable preparation reduces variability and matrix effects, enabling precise therapeutic drug monitoring and supporting informed clinical decisions. Standardized protocols also promote reproducibility across different settings, which is essential for routine clinical practice. Sample preparation techniques, mainly PPT, SPE and MEPS play a key role in assay performance. PPT is favored for its simplicity and scalability in plasma samples [38], but SPE/MEPS provides superior matrix cleanup and sensitivity, albeit with greater labor or automation demands [37].

Invasive blood sampling, primarily through serum and plasma, remains the standard in therapeutic drug monitoring for antipsychotic medications, as evidenced by its predominance across the reviewed studies. However, the invasiveness of traditional blood draws can present significant challenges for psychiatric patients, who may experience anxiety, distress, or difficulty with frequent venipuncture. This has fueled interest in non-invasive and minimally invasive sampling techniques like DBS, OF and VAMS [31,32]. Although these methods offer advantages in patient comfort, ease of collection and potential for remote monitoring, their current partial suitability reflects ongoing issues such as variability in sample quality, matrix effects and limited standardization.

For psychiatric populations, the development and validation of minimally invasive alternatives are particularly important to improve patient adherence, reduce barriers to monitoring and enable more frequent or at-home sample collection. While invasive sampling methods currently exhibit broader clinical readiness, advancing minimally invasive approaches could substantially enhance therapeutic drug monitoring by aligning analytical robustness with patient-centered care. Future research should focus on optimizing these alternative methods to achieve the accuracy and reproducibility necessary for widespread clinical adoption in psychiatric practice.

Proper sample storage is important in clinical practice to preserve the stability and integrity of antipsychotic drugs and their metabolites during transport and prior to analysis [64]. In the studies included in this review, storage temperatures varied widely, from room temperature for DBS and VAMS to −80 °C for plasma and serum, although many studies lacked full specification of these conditions. This variability in storage practices underscores the need for standardized reporting to ensure consistency and comparability in antipsychotic TDM. Inadequate storage can cause drug degradation or concentration changes, potentially compromising TDM reliability and adversely affecting clinical decisions such as dosing adjustments or adherence assessments. Thus, standardized and well-documented storage protocols, typically ranging from −20 °C to −80 °C for plasma and related matrices [65], are essential to maintain analytical accuracy and clinical relevance of UHPLC-MS/MS assays in psychiatric care.

In summary, UHPLC-MS/MS remains the gold standard for antipsychotic TDM due to its unmatched precision, recovery and matrix effect control. The choice of sample preparation must balance analytical rigor with practical considerations of laboratory infrastructure and clinical workflow. Continued optimization and standardization will facilitate broader clinical adoption, improving personalized psychiatric treatment.

Looking ahead, the integration of UHPLC–MS/MS with digital health innovations represents a promising avenue for personalized psychiatry. Telemedicine platforms and wearable sensors are increasingly used to monitor physiological and behavioral indicators, while artificial intelligence offers tools for data integration and predictive modeling. Linking these technologies with UHPLC-based TDM could enable more dynamic and patient-specific treatment strategies, fostering real-time adjustments and improved clinical outcomes.

Limitations include heterogeneity across studies, lack of standardized validation details, exclusion of non-English publications and focus on second-generation antipsychotics. Few studies linked analytical data to clinical outcomes. Standardized reporting and broader drug coverage are needed for real-world applicability. The heterogeneity of the included validation studies precluded statistical testing across biological matrices. Descriptive trends were therefore used to compare analytical performance. A further limitation of this review is the exclusion of pediatric studies. While this approach was necessary to maintain methodological consistency and ensure accessibility of included data, it may have led to the omission of relevant findings in developmental psychiatry or non-English research settings. Future systematic reviews could specifically address these populations and linguistic domains to provide a more comprehensive picture of UHPLC–MS/MS applications across diverse patient groups. Also, future comparative studies directly evaluating UHPLC–MS/MS against conventional analytical methods would further substantiate its relative advantages in specificity, sensitivity, and throughput.

Beyond analytical performance, economic considerations are also relevant when evaluating the practical implementation of UHPLC–MS/MS in routine laboratory settings.

In practical terms, the acquisition cost of a UHPLC–MS/MS system can reach into the hundreds of thousands of dollars; for example, UHPLC systems alone are priced between USD 30,000 and 150,000 depending on configuration [66]. Operational costs per sample vary: many core facilities charge between USD 15 and 80 per LC–MS/MS run. For instance, Harvard’s mass spectrometry core charges ~USD 78.37 per sample for LC-MS/MS analyses [67].

## 5. Conclusions

UHPLC–MS/MS is confirmed as the most reliable method for antipsychotic TDM, offering high recovery, precision and metabolite detection. While most studies reviewed focus on analytical method development and validation, the evidence collectively demonstrates that UHPLC-MS/MS is an effective and clinically valuable technique for therapeutic drug monitoring of antipsychotics. Furthermore, this review highlights its comparability to other analytical methods and supports its potential suitability for routine clinical practice.

## Figures and Tables

**Table 3:** Overview of UHPLC-Based Sample Preparation Techniques and Their Clinical Suitability Across Various Biological Matrices. (LLE—Liquid–Liquid Extraction, MTBE—Methyl tert-butyl ether, ACN—Acetonitrile, MeOH—Methanol, UAE—Ultrasonic-Assisted Extraction, MEPS—Microextraction by Packed Sorbent, RACNT—Reusable Activated Carbon Nanotube, DBS—Dried Blood Spot, OF—Oral Fluid, VAMS—Volumetric Absorptive Microsampling, TDM—Therapeutic Drug Monitoring, IS—Internal Standard, SPE—Solid Phase Extraction, WB—Whole Blood).

Nr.	Study	Sample Type	Sample Preparation	Clinical Context	Suitability for Standard Clinical Practice
1	Patteet et al. [40]	Serum	LLE with MTBE (pH 9.5), evaporation, reconstitution in ACN	Invasive blood samples	Yes
2	Patteet et al. [42]	Serum	Similar robust LLE method, low matrix effect	Invasive blood samples	Yes
3	Patteet et al. [41]	DBS	DBS dried 3 h, extracted with MeOH + MTBE	Non-invasive blood samples	Partial
4	Patteet et al. [43]	Oral fluid	OF buffer + LLE, evaporation, reconstitution	Non-invasive oral fluid samples	Partial
5	Patteet et al. [39]	Serum	LLE with MTBE, IS mix covering multiple antipsychotics	Invasive blood samples	Yes
6	Millan-Santiago et al. [36]	Whole blood (VAMS)	VAMS microsampling, UAE in MeOH, direct UHPLC-MS	Minimally invasive blood samples	Partial
7	Bernardo et al. [35]	DBS and WB	DBS dried overnight, WB with standard LLE	Non-invasive and Minimally Invasive blood samples	Partial
8	Toja-Camba et al. [11]	Plasma	IS spiked, ACN precipitation, quick prep	Invasive blood samples	Yes
9	Toja-Camba et al. [2]	Plasma	Same protein precipitation workflow for risperidone monitoring	Invasive blood samples	Yes
10	Cruz et al. [37]	Plasma	MEPS with RACNT, novel mini SPE, minimal prep	Invasive blood samples	Partial
11	Wang et al. [38]	Plasma	Small plasma volume, ACN or MeOH precipitation, direct analysis	Invasive blood samples	Yes
12	Pouliopoulos et al. [44]	Serum and postmortem blood	ACN + salts for protein precipitation, forensic + standard use	Clinical and forensic toxicology labs	Yes

## Data Availability

The data are contained within the article.

## Appendix A

**Table A1:** Customized Checklist Based on International Validation Guidelines. CV Coefficient of Variation, SD Standard Deviation.

1. Study Design and Reporting	
Clear description of study objectives and validation purpose	Adequate/Inadequate/Not reported
Detailed description of sample type and matrix (clinical relevance)	
Description of sample preparation procedures	
2. Reference Standards and Controls	
Use of certified reference materials or appropriate standards	
Inclusion of quality controls and blank samples	
3. Instrumentation and Method Parameters	
Detailed reporting of UHPLC instrumentation, column type, mobile phase, flow rate, detection method, etc.	
4. Analytical Performance	
Calibration curve described with appropriate concentration range	
Linearity assessed and reported (e.g., correlation coefficient R^2^)	
Sensitivity parameters reported (Limit of Detection, Limit of Quantification)	
Accuracy evaluated (e.g., recovery studies, comparison with reference methods)	
Precision assessed (intra- and inter-day variability, CV or SD)	
Specificity/selectivity evaluated (ability to separate analyte from interfering substances)	
Robustness or ruggedness testing reported (e.g., slight method variations)	
5. Sample Analysis and Validation	
Validation performed on real clinical samples, not only spiked or artificial samples	
Stability studies reported (short- and long-term, freeze-thaw, autosampler)	
Number of replicates adequate to support conclusions	
6. Data Reporting and Interpretation	
Transparent reporting of all validation results	
Discussion of limitations and potential sources of bias	

## Appendix B

**Table A2:** Analytical and Clinical Comparison of UHPLC-MS/MS and Immunoassay Methods for Risperidone, Paliperidone, Aripiprazole, and Dehydroaripiprazole Quantification.

Parameter	Risperidone and Paliperidone Toja-Camba et al. (2024) [2]	Aripiprazole and Dehydroaripiprazole Toja-Camba et al. (2024) [11]
UHPLC-MS/MS [2]		
Instrument	Xevo TQDR triple quadrupole MS	Xevo TQDR triple quadrupole MS
Internal standard	Risperidone-d4	Aripiprazole-d8
Detection transitions (*m*/*z*)	441.2→191.1 (RIS), 427.24→207.05 (PAL), 415.2→195.1 (IS)	448.2→285.2 (ARI), 446.04→285.02 (DHA), 6.3→293.07 (IS)
Calibration range	1–200 ng/mL (R^2^ = 0.99)	25–1000 ng/mL (R^2^ = 0.998)
LOD/LOQ	0.5/1 ng/mL	10/25 ng/mL
Column/temp	BEH Shield RP18, 40 °C	BEH C18, 40 °C
Flow rate/inj. Volume	0.6 mL/min, 3 μL	0.6 mL/min, 5 μL
Alinity C [11]		
Detection principle	Competitive immunoassay with photometric detection	Competitive immunoassay with photometric detection
Wavelength	604 nm	604 nm
Kit used	MyCare Psychiatry Kit for risperidone	MyCare Psychiatry Kit for aripiprazole
LOQ range	16–120 ng/mL	45–1000 ng/mL
Cross-reactivity tested	>150 drugs, RF, lipids, hemolysis	>150 drugs, RF, lipids, hemolysis
Study characteristics		
No. of samples (total)	115 plasma samples	86 plasma samples
Samples analyzed by both methods	Yes	Yes (60 samples)
Therapeutic range stratification	<20 ng/mL, 20–60 ng/mL, >60 ng/mL	<150 ng/mL, 150–500 ng/mL, >500 ng/mL
Ratio analysis	PAL/RIS ratio impact on method agreement	ARI/DHA ratio impact on method agreement
Additional analysis	-	Re-analysis after 6 months to assess reproducibility

(RIS—Risperidone, PAL—Paliperidone; ARI—Aripiprazole; DHA—Dehydroaripiprazole; IS—Internal Standard; *m*/*z*—Mass-to-Charge Ratio; LOD—Limit of Detection; LOQ—Limit of Quantification; BEH—Bridged Ethylene Hybrid; RP18—Reversed-Phase C18 Column; Alinity C—Abbott Alinity C Clinical Chemistry Analyzer).
